# Supplementary material for: Are carbon emissions trading and green financial instruments synergistic? -Comprehensive quantitative research based on content analysis
Source: PLoS One. 2024 Mar 7;19(3):e0298601. doi: 10.1371/journal.pone.0298601 (PMC10919601; doi:10.1371/journal.pone.0298601)
Supplement: S1 Data — (ZIP) [file pone.0298601.s001.zip › Data/地级市Z_F工作报告.zip!/╣≤╓▌/╫±╥σ╩╨/└┤╫╘╣1⁄2╓┌║┼í╛┬φ┐╦╩2╛▌═°í┐_2012.docx]

各位代表：

现在，我代表市人民政府向大会作工作报告，请予审议，并请列席人员提出意见。

过去五年工作回顾

过去五年，在省委、省政府和市委正确领导下，在市人大、市政协监督支持下，市人民政府坚持以邓小平理论和“三个代表”重要思想为指导，深入贯彻落实科学发展观，抢抓国家实施西部大开发、扩大内需政策机遇，实施“三新一强”战略，克服百年不遇的国际金融危机和重大自然灾害影响，圆满完成了市三届人民政府确定的目标任务。这五年，是遵义撤地设市以来经济社会发展速度最快、质量效益最好、城乡面貌变化最大的五年。

综合实力再上新台阶。主体指标实现翻番。预计全面小康实现程度为68%。初步核算，2011年完成地区生产总值1121亿元，五年年均增长14%；人均地区生产总值18000元。全社会固定资产投资814亿元，年均增长30.3%。社会消费品零售总额351亿元，年均增长20.1%。财政总收入202.2亿元，年均增长22.9%。一般预算收入84.6亿元，年均增长25.8%。城镇居民人均可支配收入17426元，年均增长14.3%。农民人均纯收入5216元，年均增长16.6%。全市金融机构各项存款余额1403亿元，增长2.8倍；贷款余额712亿元，增长2.6倍。城镇化率达37%。节能减排任务全面完成。三次产业结构比重调整为13.5：44.0：42.5，二、三产业增加值比重提高4.1个百分点。“中部崛起、西部突破、东部开发、北部攻坚”取得成效，发展态势持续向好。

“三新一强”战略取得新成效。新型工业化步伐加快。“10+工业园区建设全面推进。湘江、和平、湄潭绿色食品、桐梓循环经济等工业园区建设成效明显，仁怀名酒工业园区成为国家新型工业化产业示范基地。茅台酒新增万吨技改、构皮滩电站、遵义氧化铝、赤水竹浆林纸、桐梓煤化工、三岔拉法基水泥等项目建成投产，桐梓火电厂、绥阳煤电化、务正道煤电铝、中心城区南部企业异地技改等项目扎实推进，二郎电厂开工建设。完成规模工业增加值422亿元，年均增长15%。创新型城市建设成效明显。企业主体创新意识持续增强，创新环境不断优化。“人才工程进展顺利。市本级财政投入科技经费2862万元，年均增长47%。累计获专利授权1800件、市级以上科技成果鉴定263项；高新技术企业达35家。创建国家知识产权试点城市通过验收。获“国家商标战略实施示范城市”称号。编制完成遵义市土地利用、城市总体规划。中心城区快速扩容提质，历史文化名城保护力度加大，城市干道柔化改造全面完成，湘江河滨景观整治工程全面实施。新蒲新区、南部新城、国酒新城开发全面展开，建成共青大道、磨槽湾等骨干路网；县城建城区面积实现倍增；小城镇建设成效明显。累计完成城乡建设投资800亿元。 “四民社区”创建深入推进。荣获“国家卫生城市”、“国家森林城市”等称号；“全国文明城市”创建获提名资格，“国家环保模范城市”创建有序推进。新农村建设巩固提升。粮食年均产量稳定在310万吨左右。“八大农业产业”稳步推进。市级以上龙头企业达203家。完成村庄整治12万户，惠及48万人。创建“四在农家”示范点8200个，440万农民受益。30万农村人口脱贫。退耕还林成果得到巩固。建设营造林219万亩。森林覆盖率达48.6%。文化旅游强市建设加快。市图书馆、博物馆投入使用。建成县级文化馆及图书馆21个、乡镇综合文化站140个、“农家书屋”1742个。文化信息共享工程网络体系逐步健全。市文化艺术发展中心开工建设。全国公共文化服务示范区创建取得进展。《遵义百科全书》编纂基本完成。创作了《青山绿水红日子》等精品剧目，“十谢共产党”唱响全国。开发旅游景区（点）65个，其中新增A级旅游景区23个、省级以上旅游示范点21个。赤水丹霞列入世界自然遗产保护名录。桐梓成为世界旅游组织乡村旅游扶贫观察点。组建了遵义红色旅游集团公司。建成星级酒店45家。“中国（遵义）酒类博览会”、“中国贵州国际绿茶博览会”、“全国辣椒产业发展大会暨贵州国际辣椒节”等节会成功举办。累计接待游客7354万人次，实现综合收入603亿元，年均分别增长29.5%、39.2%。

项目建设取得新突破。完成交通投资272亿元，立体交通网络初步形成。乡、村公路通达率100%；茅台高速和习新、湄余、绥道、南楠、桐容、忠深、机场快速等二级公路通车；绥遵、龙坑至白腊坎高速公路基本建成，仁赤、杭瑞遵义段、绕城东北段等高速公路建设加快，道瓮高速公路开工。开通遵义至北京、上海、广州始发列车；黔渝快铁前期工作进展顺利。乌江、赤水河航道整治取得成效。遵义机场成功试航，茅台机场开工建设。完成水利投资56亿元。遵义灌区一期、正安石峰、仁怀石板塘等水库即将完工，道真大沙河、湄凤余灌区等工程加快推进，新蒲中桥、凤冈河头、桐梓新桥、绥阳后水河、习水铜灌口、汇川麻沟等水库开工建设。10个县小型农田水利重点建设项目启动。治理病险水库250座。实施烟水配套工程110万亩。解决221万农村人口饮水安全问题。一批大中型水电站建成投运。全市电力装机600余万千瓦。煤矿整合扎实推进，花秋、容光、兴隆等矿井建成投产。投入68亿元改造城乡电网。信息建设扎实推进。中心城区饮用水源地水质达标率100%。湄潭、绥阳、余庆、凤冈获“国家生态示范区”称号。

改革开放迸发新活力。政府机构、事业单位、行政审批、投融资、户籍、农村集体林权、文化体制等改革稳步推进。遵渝战略合作全面开启，与重庆大渡口、上海杨浦等地结为友好城市，与中电投、中建、中石化、中石油、保利等央企合作加强，政银、政企、政校及区域合作全面展开。招商引资累计到位资金1600亿元，年均增长77%。遵义海关开关运行。累计完成进出口总额10亿美元，年均增长23%；累计实际利用外资2亿美元，年均增长83.8%。国家级遵义经济技术开发区，娄山关、仁怀、红花岗、湄潭、赤水、绥阳及遵义县苟江、和平省级经济开发区获准设立。非公有制企业达7175个，从业人员19万人，经济总量占地区生产总值比重接近50%。

和谐遵义呈现新面貌。新增城镇就业25万人、下岗失业人员再就业6.5万人，城镇登记失业率3.56%；农村劳动力转移就业63万人。城乡居民最低生活保障实现全覆盖。88万人次享受城乡医疗救助。20余万人次享受临时救助。改造农村危房11.4万户、城市棚户区1.29万户；新建廉租住房1.97万套、公共租赁住房2425套、经济适用住房1万余套。城镇低收入家庭住房保障制度基本建立。水利水电工程移民搬迁安置6500人。完成农村异地扶贫安置1.2万人。深化医药卫生体制改革完成阶段性目标，公立医院改革试点取得进展。市第一人民医院成功创建“三甲”，县级人民医院基本达到“二甲”水平。城镇居民医保、新型农村合作医疗实现全覆盖，城镇职工医保实现市级统筹。国家基本药物制度全面推行，公共卫生服务均等化提质扩面。重大疾病防控、食品药品监管、卫生监督、血液工作得到加强。人口自增率控制在5.5‰以内，低生育水平持续稳定。价格调节基金补贴制度进一步完善。投资10.6亿元实施各类教育工程1035项。小学适龄儿童入学率99.9%，初中适龄少年入学率97.4%，青壮年非文盲率99.99%。“两基国检”顺利通过。省级示范性普通高中达19所，多项高考指标居全省前列。高校服务地方发展能力明显增强。幼儿教育、特殊教育、职业教育、民办教育健康发展。建成市民体育健身中心等场馆。奥运会、民运会火炬在遵顺利传递。遵义籍运动员实现中国奥运拳击和贵州奥运金牌零的突破。建成广播电视“村村通”38万户，广播、电视覆盖率分别为93.8%、95.2%。连续六次获“全国双拥模范城”称号。“平安遵义”建设取得成效。“五五”普法圆满完成。人民调解、民主法治建设扎实推进，信访维稳、应急处置、部队和国防后备力量建设得到加强。社会治安防控体系逐步完善，公众安全感不断增强。蝉联全国社会治安综合治理“长安杯”称号。安全生产事故起数、死亡人数连续11年实现“双降”。民族宗教、机构编制、外事侨务、人防战备、水文、气象、地方志、档案、妇女、儿童、供销社、公积金、慈善、老龄人、残疾人、红十字等事业全面发展。

行政效能得到新提升。学习实践科学发展观、创先争优、“三个建设年”、“四帮四促”等活动扎实开展。实施中心城区土地“五统一”、项目建设“三基本”等措施，发展难题不断破解。行政复议试点工作有效加强，行政处罚自由裁量权不断规范。实施简政放权，减少、合并行政许可事项339项、非行政许可审批事项119项，减少市级收费项目333项。开通网上行政审批和电子监察系统。政务服务中心“两集中、三到位”扎实推进。自觉接受市人大、市政协监督，累计办理人大代表建议666件，政协提案1418件，办结率为100%，满意和基本满意率为99%。行政监察、审计监督和行政问责力度加大，政府廉政建设得到加强。

各位代表，过去的五年是极不平凡的五年！面对各种困难与挑战，我们团结奋进，克难攻坚，各项工作取得了令人振奋的成绩。这些成绩的取得，得益于省委、省政府和市委的坚强领导，得益于市人大、市政协的监督支持，得益于全市干部群众艰苦卓绝的共同努力。在此，我代表市人民政府，向全市各族人民、广大干部职工，向各民主党派、工商联、人民团体、无党派人士，向驻遵解放军指战员、民兵预备役人员、武警和消防官兵、公安政法干警，向历届老领导老同志及关心支持遵义经济社会发展的各界人士，表示衷心的感谢并致以崇高的敬意！

各位代表，过去五年是值得认真总结的五年！通过五年的实践，我们深切感受到，推动遵义经济社会提速赶超、转型跨越，必须始终坚持发展率先，以科学思路指导实践，千方百计推动跨越发展；必须始终坚持思想领先，以新观念开启新思路，千方百计推动创新发展；必须始终坚持开放争先，以自身优势引资引智，千方百计实现借力发展；必须始终坚持民生优先，以惠民举措凝聚力量，千方百计增加群众福祉；必须始终坚持务实为先，以优良作风提供保障，千方百计提升行政效能。

在肯定成绩的同时，我们也清醒地看到，在经济社会发展中还存在一些矛盾和问题，主要体现在：经济总量不大，发展速度不够快；经济结构不够优化，城乡二元结构和区域发展不平衡问题还较突出；干部思想不够解放，执行能力还需加强；发展环境有待改善，政府职能还需进一步转变。这些矛盾和问题，我们将通过发展的办法加以解决。

今后五年政府的主要工作

当前，遵义已进入全面建设小康社会新的发展阶段，既处于工业化、城镇化、农业现代化的加速发展期，又处于经济社会实现增比进位突破的战略机遇期。从外部环境看，国家实施新一轮西部大开发战略，制定黔中经济区、武陵山区域发展规划；出台支持贵州又好又快发展的政策措施，加快建设黔北经济协作区，构建连结成渝经济区和黔中经济区的经济走廊；省委、省政府支持遵义建设统筹城乡发展试验区、成渝经济区产业扩散转移重要辐射区、贵州发展“金三角”重要一极。从自身发展看，全市综合实力不断增强，发展速度不断加快，具备了“提速赶超、转型跨越”的基础条件；广大干部群众思发展、盼发展、谋发展的愿望更加迫切。这些机遇和条件，正是我们加快发展的信心和决心所在。

今后五年政府工作的指导思想：高举中国特色社会主义伟大旗帜，以邓小平理论和“三个代表”重要思想为指导，深入贯彻落实科学发展观，把贯彻落实《国务院关于进一步促进贵州经济社会又好又快发展的若干意见》作为全市工作的总抓手，按照“两加一推”主基调和“增比进位突破”、打造贵州发展“金三角”的新要求，突出“提速赶超、转型跨越”主旋律，大力实施“开放开发、改革创新、三化同步”主战略，全面加强经济、政治、文化、社会和生态文明建设，加快实现黔北提升，为把遵义建设成为宜居宜业宜游的现代化特色城市，在全省率先实现全面小康而努力奋斗。

今后五年政府工作的主要目标：到2016年，全面小康实现程度达85%以上；完成地区生产总值2400亿元，年均增长17%以上；人均地区生产总值突破35000元；全社会固定资产投资累计完成10000亿元；社会消费品零售总额870亿元，年均增长20%以上；财政总收入500亿元，年均增长20%以上；城镇居民人均可支配收入35200元，年均增长15%以上；农民人均纯收入10200元，年均增长16%以上；人口自增率控制在5‰以内；城镇化率44%以上；城镇登记失业率控制在4.5%以内；单位生产总值能耗、主要污染物排放总量控制在省下达指标内。

围绕上述目标，我们重点抓好以下工作：

一、坚定不移建园区聚产业，强力推进新型工业化

集中力量建设产业园区。加速基础设施建设。优先保障投入，产业园区财税收入市县留存部分全部用于滚动发展；将基础设施贷款纳入财政偿债机制，支持园区、高新技术项目争取贷款。优先保障用地供给，坚持土地指标向园区倾斜，产业园区土地收益全部用于园区基础设施建设和土地开发。优先保障供电，力保园区企业不拉闸、不限电。优先保障供水，大力实施园区发展需要的骨干水源、配套供水管网工程。完善机制推动园区发展。推动开发区与产业园区建设紧密融合，规划建设产业特色鲜明、综合配套能力强的产业集聚区。理顺园区管理体制。健全考核评价体系，完善园区建设、退出机制。到2016年，湘江、和平、仁怀名酒、汇川机电工业园区年产值分别达500亿元以上；娄山关经济开发区年产值超200亿元；湄潭经济开发区、绥阳风华工业园区及遵义县苟江冶金、桐梓煤电化、务正道煤电铝等循环经济工业园区，年产值分别达100亿元以上；加快建设一批年产值超10亿元的工业园区；全市工业增加值年均增长25%以上。

加快发展特色优势产业。依托资源条件和工业基础，加快建设西部乃至全国重要的能源、资源深加工、装备制造业、特色轻工业和新材料基地。扎实推进务正道煤电铝、桐梓火电厂、桐梓煤化工二期、习水煤化工、绥阳煤电化、40万吨铝精深加工、二郎电厂、赤习油气、风电、新增2000万吨煤矿等资源开发项目。全力推进新增2万吨茅台酒、电解铝厂升级、长征电气系列企业技改、遵宝钛业万吨海绵钛深加工、千万吨新型干法水泥、赤天化纸业10万吨竹纤维技改等产业提升项目。加快贵州钢绳、遵义钛业、遵义氯碱、遵义汇兴铁合金、○六一基地在遵企业、遵义卷烟厂等企业异地搬迁技改。培育一批百亿级大企业，推动企业上市。到2016年，白酒产量达60万千升以上，打造茅台酒“世界蒸馏酒第一品牌”取得重大进展；白酒、材料、能源、机电及装备制造业分别实现年产值1000亿元以上；化工、特色食品产业分别实现年产值200亿元以上；烟草、制药、竹业、新兴产业分别实现年产值100亿元以上。

着力提升工业发展质量。积极利用高新、信息技术，改造提升传统产业，推进工业和信息化融合。培育发展电子信息、新材料、新能源、生物工程、节能环保、软件和服务外包等新兴产业。积极推动煤电铝、煤电化、煤电冶等一体化发展。强化节能减排和资源综合利用，加快淘汰落后产能，大力发展循环经济。到2013年，工业用水重复利用率达90%以上，重点工业污染源废水排放达标率100%。

二、坚定不移扩新区提品质，加速推进绿色城镇化

高起点完善城市规划。完成经济开发区、中心城区综合交通和山体公园规划，以及县城新区、工业园区控规和乡镇总规编制。强化规划执行，整治违法建设。推动绥阳、遵义县撤县设区，桐梓、湄潭撤县设市。到2016年，中心城区建城区面积150平方公里以上、人口150万以上，形成承载200万人口城市框架；县城所在地分别承载20万人以上；中心镇、重点镇分别承载5—10万人。

高强度推进城镇建设。加快推进新蒲新区开发。完善新火车站片区、礼仪坝片区、空港片区城市路网。建成遵义医学院、遵义师院、遵义医专、遵义干部学院、遵义职院、遵义四中等新校区。2013年实现市级行政中心搬迁。建成奥体中心、会展中心等一批城市基础设施。全面推进城市综合体建设。加快完善南部新城、国酒新城及各县城新区基础设施。建成汇川大道延长线、遵龙快线北段、汇川至绥阳及新蒲至湄潭黄家坝城市交通干道、赤水30里河滨大道。继续实施主城区交通疏解、棚户区改造、红色文化展示提升等工程，完善学校、医院、市场、垃圾无害化处理等功能性基础设施。新增城镇森林公园100平方公里。加强历史文化名镇建设与保护。加快推进茅台镇周边环境整治，争取用5—10年时间，将茅台镇打造成“中国国酒之心”，将仁怀市打造成 “中国国酒文化之都”。集中打造习水土城华润希望小镇等一批富有黔北特色的小城镇。到2016年，累计完成住房和城乡建设投资3500亿元以上。

高标准建设宜居城市。加大公共设施投入。加快数字遵义地理空间框架建设，大力推进城镇规划、国土、交通、治安、应急等信息资源共享，实现中心城区城市管理信息化、精细化和全时段、全方位覆盖。合理配置资源，大力发展公共交通。巩固“国家园林城市”、“国家森林城市”、“国家卫生城市”创建成果，加快“国家环保模范城市”创建，确保2014年“全国文明城市”创建成功。实行最严格的耕地、环境保护制度，保障可持续发展。继续推进乌江、赤水河流域水环境综合治理。到2016年，基本解决集中式饮用水源安全问题，中心城区、85%以上的县城环境空气质量优良率达90%以上，全市森林覆盖率提高到50%以上。

三、坚定不移强优势兴龙头，着力推进农业现代化

大力调整农业结构。巩固退耕还林成果，推进烟草、蔬菜、茶叶、中药材和畜牧业发展。加快遵义山区现代农业示范区建设。到2016年，完成退耕还林50万亩、封山育林99万亩；商品蔬菜面积达150万亩，辣椒200万亩，高粱200万亩，茶叶150万亩，中药材150万亩；粮食年产量稳定在300万吨左右；年收购烤烟200万担左右；着力培育茶叶、辣椒、生态畜牧业等年综合产值分别超100亿元的主导产业。

大力培育龙头企业。强化农业项目招商，鼓励龙头企业加大投入，在农产品精深加工、品牌创建和附加值提高上实现突破。规划建设一批现代农业示范园区。积极发展冷链物流服务。推动专业市场建设。大力发展无公害农产品、绿色食品、有机食品、地理标志保护产品，打造“放心食品”生产示范区。到2016年，引进培育市级以上龙头企业500家，培育销售收入超亿元的龙头企业30家。

全力实施扶贫开发。抓住武陵山、乌蒙山扶贫攻坚规划实施机遇，坚持政府主导，分级负责，突出重点，合力推进。将符合条件的贫困人口全部纳入农村最低生活保障。加快重点县、乡镇“减贫摘帽”步伐，推动贫困地区农民人均纯收入增幅高于全市平均水平，基本公共服务主要指标接近全市平均水平，努力实现扶贫对象“两不愁、三保障”的目标。

深化新农村创建内涵。加强社会主义新农村建设，促进基础设施向县以下延伸。大力开展村庄整治，扩大“四在农家”覆盖面。以高速公路、铁路、国省干道沿线为重点，突出特色，挖掘内涵，全力打造在西部地区乃至全国具有重要示范带动效应的景观路、产业路、生态路。到2016年，实现“四在农家”创建点全覆盖。

四、坚定不移抓招商建平台，深入推进开放开发

更大力度开放合作。按照“共建黔中、融入成渝、连通闽粤、伸向沪宁”思路，构建全方位、宽领域、多层次的开放开发格局。提速遵义国家级经济技术开发区和省级经济开发区建设，加快娄山关国家级高新技术产业开发区申报步伐。加强与泛珠三角、长三角、环渤海经济圈及友好城市、对口帮扶城市经贸交流。鼓励支持临边县市与成渝经济区加强合作。建立健全安商、扶商机制。创新招商引资方式，充分发挥商会及政府驻外机构作用，加大环境扶商、产业招商、园区招商、以商招商、企业招商、大项目招商力度，提高资金到位率和项目落地率。确保到位资金年均增长45%以上。

全力壮大民营经济。认真贯彻落实加快发展民营经济的政策措施，降低准入门槛，优化发展环境，在税收、金融、土地等方面加大扶持，增强综合竞争力和可持续发展能力。用3年左右时间，实现民营经济主体、总量、就业、创新能力倍增目标。

加快发展对外贸易。围绕资源优势，重点培育骨干企业，加强出口品牌建设，大力推进酒类、机电、农产品、IT配套产业出口基地建设。努力建成具有保税功能的加工贸易聚集区。加快建设遵义“无水港”和对外开放口岸。建成国家级高低压电器检测中心、绿茶检测中心。到2016年，外贸进出口总额完成6亿美元，年均增长20%以上；实际利用外资3亿美元，年均增长35%以上。

五、坚定不移强统筹增活力，稳步推进改革创新

统筹城乡综合改革。总结推广湄潭农村改革和凤冈“三资转换”经验，完善土地承包经营权，支持农民依法自愿有偿流转土地，推行城乡建设用地增减挂钩。探索推进集体林权、土地使用权、宅基地、房产等贷款抵押担保，着力在破解农村发展投入上取得突破。加快遵义统筹城乡发展试验区建设，积极申报全国农村综合配套改革试验区。

推进重点领域改革。创新国有资产经营管理方式，整合优质资源，做强城建、交通、工业、旅游、水务等国有控股投融资平台，增强投融资能力。支持组建茅台集团财务公司。继续深化户籍、医药卫生、文化、收入分配、供销合作社等改革。积极稳妥推进事业单位分类改革。深化财政体制改革，完善偿债机制，增强市级调控能力，进一步调动县级积极性。推动资源性产品价格改革。深入贯彻落实《省委、省政府关于进一步加快发展县域经济的意见》，推动扩权强县改革。建立健全与事权相匹配的行政管理体制。科学制定县域综合考评体系。出台加快北部攻坚、东部开发及支持新蒲新区、遵义经济技术开发区发展的具体措施。

提高科技创新能力。加快推动创新型城市建设。继续实施“15851”人才工程。切实解决人才紧缺与人力资源浪费并存的问题，用活用足各类人才。积极创造条件，吸引高层次人才来遵创业发展，建立持续稳定的专业人才引进机制。推进专利权、商标权质押。确保市本级研发经费投入增速不低于一般预算收入增速。突出企业创新主体，促进政、产、学、研、用紧密结合，提升科技创新能力。到2016年，省级以上重点实验室、工程技术中心、院士工作站等创新平台达20家，专利申请量6000件、授权3600件，高新技术企业70家以上。

六、坚定不移夯基础破瓶颈，快速推进交通水利电力信息设施建设

加速交通设施建设。完成交通投资750亿元以上。建成仁赤、余凯、杭瑞遵义段、绕城、道瓮、绥正、务正、余花等高速公路，推动成都至遵义、赤水经正安至沿河、赤水经罗甸至百色、凤冈至武隆等高速公路建设。加快推进县际公路建设，着力建成一批跨区域连接路、旅游路、产业路。建成遵义旅游客运中心、颜村综合公路客运枢纽和高桥、新蒲公路客运站。切实做好道路交通保畅。推动维修改造326国道遵义段。加快推进茅台机场建设，开展黔北机场前期工作。配合做好昭黔铁路遵义段、黔渝快速铁路遵义段等建设工作。加快乌江构皮滩水电枢纽通航设施及赤水河航道建设。到2016年，高速公路通车总里程突破1000公里，公路总里程突破3.3万公里，实现县县通高速公路、村村通油路、重点景区通油路目标；航道总里程突破900公里；民航、快速铁路实现零的突破。

加快水利电力建设。抓住国家专项支持贵州实施水利、生态、石漠化“三位一体”项目机遇，着力解决工程性缺水问题。实施习水东风、仁怀怀南、正安庙塘等大中型灌区续建配套与节水改造工程。开工建设中型水库19座、小型水库51座。力争开工建设赤水金龙等水库。治理病险水库158座、中小河流60条。继续实施“五小”工程。基本解决农村饮水安全问题。实现人均半亩口粮田目标。着力缓解城镇防洪压力。进一步理顺水利管理体制。落实好水利水电工程移民安置和后期扶持政策。投资107亿元，完善输变电网设施建设，形成以500千伏变电站为支撑、220千伏电网为骨架的城乡输配电网络。

推动信息网络设施建设。积极推进“三网融合”，建设无线城市。提高电话、互联网普及率，力争行政村基本通宽带，已通电的20户以上自然村和重要交通沿线通信信号基本覆盖。加快完善公共服务领域信息化建设。实施电子政务提升工程和“三农”信息服务工程。强化信息网络安全与应急保障基础设施建设。加快邮政普遍服务体系建设。

七、坚定不移铸精品提内涵，大力推进第三产业发展

深入推进宜游城市建设。围绕打造“中国最佳旅游城市”目标，突出“红色遵义、绿色家园”特色，深度打造“六大旅游”品牌。加强历史文化名镇及旅游资源富集城镇保护和建设。大力发展红色旅游，加强以遵义会议纪念体系为重点的经典景区基础设施建设。加快余庆飞龙湖、湄潭乡村旅游、凤冈生命产业特区、桐梓黄莲、道真大沙河、正安九道水、习水生态旅游产业园区、新蒲沙滩、遵义县苟坝会议及汇川海龙囤古军事城堡、国际温泉城、娄山关红色旅游等景点开发建设。推动统一开发赤水河流域旅游资源。支持赤水建设国际休闲旅游目的地。快速拉动以高档酒店、度假型酒店、乡村旅馆、旅游地产、旅游商品开发等为重点的配套设施建设。到2016年，新增A级旅游景区（点）22个、省级以上休闲农业与乡村旅游示范点11个；新增星级酒店23家以上，其中中心城区五星级酒店10家以上；旅游从业人员25万人以上，接待人次、综合收入年均分别增长30%、35%以上。

加强国家公共文化服务体系示范区建设。深入贯彻落实十七届六中全会精神，推动多民族文化大发展、大繁荣，努力实现城乡居民基本公共文化服务均等化。建成市文化艺术发展中心、工人文化宫、大剧院、美术馆、档案馆，新建汇川、仁怀、赤水文化馆，汇川、遵义县、湄潭、仁怀图书馆，贵州清早期工业文明博物馆、习水博物馆。新建、改扩建14个县级档案馆。深入开展“五心教育”等主题实践活动。广泛开展群众性精神文明创建和志愿者服务活动。发展红色文化、酒文化、茶文化、民族民间文化、文化创意、动漫等产业，打造一批文化产业集团、文化产业园区和精品影视剧。到2016年，文化产业产值达160亿元。

提速第三产业发展。着力打造现代服务业，拓展生产性服务业领域，丰富消费性服务业内涵，壮大提升第三产业。加快发展金融服务业。规划建设中心城区大型商场和专业市场，重点打造龙坑、忠庄专业市场，积极发展礼仪、高桥、忠庄、南白等新兴商圈。加快建设遵义航空港、铁路货场和公路运输等物流大通道。启动李家湾、南宫山、空港物流园区建设。建设仁怀中国名优白酒交易中心、绥阳绿洲农产品商贸中心。做大做强虾子辣椒批发市场。建成“农家店”3000个。全面完成标准化市场建设改造。第三产业增加值年均增长15%以上。

八、坚定不移谋民利惠民生，全面推进社会管理创新

扎实推进宜业城市建设。以建设国家级创业型城市为载体，加快建设创业孵化园、大学生创业园、农民工创业园，推动全民创业。强化职业技能培训，抓好实训基地建设，完善公共就业服务和就业援助制度，建立统筹城乡的人力资源市场。大力实施增岗行动计划，做好高（职）校毕业生就业、复员转业军人安置就业和企业下岗职工、失业人员、零就业家庭就业工作，促进农民和城镇无业人员转移就业。确保城镇新增就业25万人以上。

加快发展社会事业。突破教育瓶颈。实施学前教育工程251个、义务教育阶段工程2180个、高中阶段教育工程27个。加快发展学前教育，学前三年入园率达65%以上。着力解决进城务工人员子女入学问题；义务教育巩固率达95%。突破高中阶段教育，实现县县有省级示范性普通高中目标，高中阶段毛入学率达65%以上。加快发展高等教育。继续推动遵义大学、中国遵义干部学院筹建工作。大力发展职业教育。鼓励并依法规范民办教育发展。加强教师队伍特别是农村中小学教师队伍建设。构建公共卫生服务体系。继续推进公立医院改革，支持组建公立医院投融资集团。积极推进城乡医疗保险资源整合。深入实施国家基本药物制度。推动市第二、第三人民医院和遵义县人民医院达“三甲”水平，其它县级人民医院全面达“二甲”水平。加强疾病防控、卫生监督、妇幼保健、职业病防治、食品药品监管、卫生应急处置、血液保障等能力建设。加强人口计生工作。坚持“利益导向从优、依法管理从严”原则，提升人口计生工作水平。到2016年末，常住人口符合政策生育率达97%，出生人口性别比回归正常范围。加快发展其他各项社会事业。建成城南体育中心、168个村级农民体育健身工程、122个社区全民健身路径工程。开展地面数字电视工程试点，巩固提升广播电视覆盖率。加强部队和国防后备力量建设。依法管理宗教事务。促进民族地区发展。完成《遵义市志》编纂。推动机构编制、外事侨务、人防、水文、气象、妇女、儿童、慈善、红十字等事业全面发展。

着力提升保障能力。健全社会保障体系。扩大社会保险覆盖面。全面推行城乡养老保险。推进残疾人社会保障及服务体系建设。建立健全城乡低保动态管理机制。完善临时救助、大病救助制度。加强乞讨、流浪、精神病人救助。提升住房保障水平。加大保障性住房建设力度，基本解决城镇低收入家庭住房困难。全面完成农村危房改造任务。逐步提高住房公积金缴存比例和基数。增加城乡居民收入。建立健全职工工资正常增长机制。加强城乡居民生活必需品价格监测，严厉打击价格违法行为，建立健全低收入群体价格临时补贴与物价上涨联动机制。

打造平安和谐遵义。扎实开展“六五”普法，推进依法治市进程。以推进“平安遵义”创建为载体，加强社会治安综合治理，依法打击违法犯罪行为。加强刑释解教人员、问题青少年等特殊人员帮教管理。畅通信访渠道，着力构建大信访格局。深入推进“四民社区”、“农村社区全覆盖”创建工作。加强民主法治建设。完善社会信用体系，建立健全守信受益和失信惩戒制度。坚持安全第一、预防为主、综合治理，加大投入，建立完善县、区（市）长安全发展承诺制、季度通报制，开展重点行业和领域隐患治理，确保安全生产形势持续稳定好转。打造平安和谐的社会环境，人民群众安全感达90%以上。

九、坚定不移转作风提效能，扎实推进政府自身建设

解放思想，敢为人先。大力倡导敢闯敢试、敢于担当、敢于负责的精神，只要符合科学发展观要求，只要有利于遵义发展，只要为百姓谋福利，就要敢于突破常规、敢于适度负债、敢于大胆让利，不怕不理解、不怕遭指责，鼓励探索，宽容失败，营造想干事、能干事、干成事的发展环境。

转变作风，真抓实干。继续发扬干字当头、拼字求胜的作风，用各级干部的辛苦指数换取群众的幸福指数。坚持重心下移、力量下沉，相信基层、体谅基层、服务基层。加强调查研究工作。继续推进执行力建设，严格落实“四要十不准”，确保政令畅通、令行禁止，确保定下的目标一抓到底、部署的工作抓出成效。切实改变会风文风，着力解决会议多、文件多、应酬多、不在状态的问题，促进各级干部把所有心思用在事业上，把全部精力用在工作上，把最大劲头用在发展上，凝聚“遵义力量”、创造“遵义速度”、提升“遵义形象”。

依法行政，清正廉洁。按照建设法治政府、服务政府、责任政府、廉洁政府的要求，着力提升政府公信力，努力建设人民满意的政府。修订完善《政府工作规则》。加强政务环境建设，继续推进政务公开。自觉接受人大、政协监督，认真办理建议、提案。全面落实党风廉政建设责任制，严格工程建设招投标和监理程序，加强项目监管和预算管理，推进源头治腐。加强政府采购、行政监察和审计监督，严肃查处违法违纪案件，促进政府廉政建设。

2012年经济社会发展主要目标任务

2012年，是新一届政府的开局之年。我们将紧紧抓住国家扩大内需、发展实体经济的政策机遇，牢牢把握“稳中求进”、“提速转型”总基调、总目标和“稳中求快、快中保好、能快则快、又好又快”总要求，深入贯彻落实《国务院关于进一步促进贵州经济社会又好又快发展的若干意见》，重点抓好宣传学习，重点抓好实施意见研究制定，重点抓好专项规划编制和试点示范工作方案制定，重点抓好重大问题调研，重点抓好与中央及省有关部门对接，重点抓好项目化落实，使中央政策尽快转化为支撑遵义发展的现实生产力，走出一条符合遵义实际和时代要求的追赶型、跨越式后发赶超之路。

主要预期目标：完成地区生产总值1366亿元，增长17.5%；全社会固定资产投资1302亿元，增长60%；规模工业增加值544亿元，增长25%；社会消费品零售总额421亿元，增长20%；财政总收入253亿元，增长25%；一般预算收入106亿元，增长25%；城镇居民人均可支配收入20040元，增长15%；农民人均纯收入6050元，增长16%；城镇化率达39%；城镇登记失业率控制在4.2%以内；人口出生率、自增率分别控制在10.5‰、5.1‰以内；安全生产事故起数和死亡人数控制在省下达指标内；节能减排完成省下达任务。

围绕上述目标任务，我们着力抓好以下工作：

着力推进园区建设。实现园区基础设施“七通一平”目标。完成工业投资550亿元，建设重点项目115个以上。长征风电、汇川海螺水泥等项目建成投产，加快二郎电厂、绥阳煤电化及贵州钢绳、遵义碱厂、遵义汇兴铁合金等企业异地技改，推进务正道煤电铝一体化项目建设，开工建设务川80万吨氧化铝、○六一基地在遵企业异地技改等项目。加大白酒技改和资产整合力度。支持组建“大茅台集团”。加快仁怀名酒工业园区建设。申报“仁怀酱香型白酒”地理标志。完成茅台酒新增2600吨技改。实现规模以上白酒产量28万千升。推动遵义烟厂异地技改，力争完成卷烟产销55万大箱。落实电煤供应新机制。推动煤矿兼并重组，建成一批大中型矿井。保障成品油气等重点物资供应稳定。

着力完善城镇基础设施。完成城镇及房地产投资590亿元以上。全面推动城市综合体建设。大力推进汇川大道延长线、高新快线、遵龙快线北段等城市干道建设。建成东南大道。继续实施中心城区交通疏解工程。启动中心城区东联线改造。加快推进遵义医学院、遵义职院和遵义四中分校新校区建设。开工建设中建四局遵义商务中心、奥体中心、新闻中心、会展中心、青少年活动中心、规划展览馆，启动遵义干部学院、遵义师院、遵义医专搬迁工程。加快推进新舟坝、高桥等棚户区改造和湘江河滨景观整治。加大城市园林景观规划建设力度。深入推进“整脏治乱” 。加大违法建设打击力度。确保“国家卫生城市”通过复检验收，“全国文明城市”、“国家环保模范城市”创建实现年度目标。全面运行中心城区数字城管信息平台。建成中心城区南部污水处理厂、青山供水工程、县城垃圾无害化处理工程，启动中心城区东部垃圾填埋场工程。

着力发展现代农业。落实“米袋子”、“菜篮子”工程责任制。粮食总产量稳定在300万吨左右，种植商品蔬菜100万亩、辣椒150万亩、高粱80万亩。新建改造茶园15万亩、竹10万亩，新增中药材20万亩、干鲜果40万亩。收购烤烟190万担以上。实现生态畜牧渔业产值80亿元。新增市级以上龙头企业50家。农业综合机械化水平达25%。建成10个高标准现代烟草农业基地单元。启动实施第二期天保工程公益林建设，完成营造林70万亩。继续实施“集团帮扶”。实现1个重点县、20个重点乡（镇）“减贫摘帽”，减少贫困人口15万人。广泛开展小康示范型、巩固提高型、推广普及型“四在农家”创建。

着力夯实发展基础。完成交通投资120亿元以上。遵义机场全面通航。加快茅台机场和在建高速公路建设。开工建设绕城北环、务正高速公路。启动机场、绥正、余花高速公路前期工作。加快遵义公路旅游客运中心及高桥、新蒲公路客运站等交通枢纽建设。做好黔渝快铁协调服务。加强乌江等级航道、水上交通安全监管救助中心及乌江渡、楠木渡港口建设。完成水利投资25亿元以上。加快推进在建水利工程，开工建设务川岩溪、仁怀大沙坝等水库，力争正安八岔林、湄潭关坎脚等水库开工建设，启动湄凤余灌区四期、遵义灌区续建配套工程。完成50座病险水库除险加固，治理中小河流22条、水土流失面积240平方公里。新增、恢复、改善灌溉面积35万亩。启动道真桷木塘、官庄和习水兰子口水电站建设。完成城乡电网改造投资26.5亿元。实现农村电网改造全覆盖。进一步理顺农村电网管理体制。推进“三网融合”，积极建设“无线城市”。

着力推进开放开发。继续推动渝遵战略合作。积极组织赴重庆开展大型招商引资活动。继续面向长三角、珠三角等地区招商。确保招商引资到位资金1407亿元，增长50%。加快出口加工区申报和“无水港”、对外开放口岸建设。完成进出口总额3亿美元，增长30%。引进外资总额2亿美元，增长30%以上。清理压缩行政审批项目20%。实施“三年倍增计划”，落实好兴办、领办微型企业“3个15万元”的鼓励措施，确保民营经济增长27%以上。

着力推进改革创新。深化农村各项改革，加快统筹城乡发展试验区建设，推进农村集体土地使用权流转、集体林权制度和供销社体制改革。加快投融资、国有资产管理改革。推进财税和水、电、气等价格改革。启动实施邮政储蓄银行、农村合作金融机构、村镇银行乡镇全覆盖工程。加快国有企业改革。深化文化体制改革，加快国有经营性文化单位转企改制。深化以公立医院为重点的医药卫生体制改革。积极推动开发区、新蒲新区管理体制改革。加快行政管理和事业单位绩效工资制度改革。组织实施国家和省级科技项目210个。

着力壮大县域经济。更加突出区域统筹，落实中部崛起、西部突破、东部开发、北部攻坚区域发展规划，健全完善统筹区域发展的体制机制。加快经济强县强镇建设。全力推动增比进位，仁怀尽力保位；赤水、红花岗、桐梓实现综合排名进位；汇川、遵义县、湄潭、道真综合排位及地区生产总值增速在全省进5位以上，余庆、凤冈、务川、正安、绥阳、习水进10位以上。

着力提升三产总量。加快国家公共文化服务体系示范区创建。建成市文化艺术发展中心，汇川文化馆、图书馆、档案馆，仁怀文化馆、图书馆，赤水文化馆，红花岗档案馆。推动群众文化广泛开展。启动土司文化博物馆和文化产业园区规划建设。提升“六大旅游”品牌吸引力。开工建设遵义会议陈列馆新馆，打造红色文化主题公园，做大做强遵义红色旅游集团公司。加快凤冈生命产业特区、桐梓黄莲等景区开发，发展集旅游、文化、休闲、度假、居住为一体的旅游地产。接待游客3000万人次以上，完成综合收入280亿元以上。加快市场体系建设。出台措施促进物流园区加快发展。推进遵义商贸城、农产品和小商品交易中心等专业市场建设。开工建设会展中心。承办好贵州省第二届农民运动会等节会活动。开工建设一批星级酒店，新长征大酒店等建成投用。积极实施“引银入遵”工程，推动中信、民生、招商、重庆等银行在遵设立分支机构。培育壮大农村金融机构。繁荣城乡消费市场。加快发展信息、社区服务等面向生产、面向民生、面向群众的服务业。推动住宿、餐饮、交通运输等传统服务业转型升级。第三产业增加值增长16%以上。

着力改善和保障民生。重点办好“十八件实事”：⑴培训农村劳动力2万人，转移农村劳动力8万人，城镇新增就业5万人以上。⑵对全市71.64万因灾需口粮救助的低保对象和缺乏自救、互救能力的困难群众进行冬春救助。⑶城乡居民最低生活保障标准提高10%以上。城市低保月平均保障标准从279元提高到306元，农村低保年平均保障标准从1468元提高到1615元。⑷实现新型农村和城镇居民社会养老保险全覆盖，确保60周岁以上符合参保条件的农村居民和城镇未就业居民应保尽保。⑸完善城镇居民医保和新型农村合作医疗制度，政府补助标准提高到每人每年240元。新农合政策范围内住院费用报销比例从70%提高到75%，政策范围内统筹基金最高支付额由5万元提高到10万元。⑹实施生态移民工程，搬迁移民7000人。⑺新建敬老院70所。⑻50万名农村义务教育阶段学生享受“营养午餐”。⑼建设农村寄宿制学校学生宿舍10万平方米、教师周转宿舍7.3万平方米，新建、改扩建乡镇公办幼儿园92所，竣工薄弱学校改造项目60个，实施高中阶段教育项目24个。⑽推进乡村卫生服务一体化管理，对12所中心乡镇卫生院进行达标改造。⑾提高村医收入水平，财政补助从每月200元提高到300元，年终一次性奖励增加到1200元。⑿加快推进市第一、第四、第五人民医院和遵医附院新蒲医院建设，完成市第二、第三人民医院综合楼建设。⒀实施农村危房改造5.7万户，新开工城镇保障性住房6910套、棚户区改造住房10000套。⒁新增解决53万农村人口饮水安全问题。⒂完成通村油路1300公里。⒃新建及改造35千伏线路1725公里、10千伏线路3720公里、低压线路1.23万公里。⒄新增118个以上自然村通电话、55个行政村通宽带。⒅建成3.2万座农村直播卫星地面接收站。

各位代表，发展的航船风举帆张，肩负的使命更加艰巨。让我们紧密团结在以胡锦涛同志为总书记的党中央周围，在省委、省政府和市委的坚强领导下，团结和依靠全市各族人民，奋力拼搏，励精图治，扎实工作，全力推动经济社会又好又快、更好更快发展，以优异的成绩迎接党的十八大和省第十一次党代会胜利召开！
